# Supplementary material for: Nup42 safeguards heat-induced mRNAs from nuclear condensation to support chaperone synthesis
Source: bioRxiv. 2026 Feb 11:2026.02.10.705189. Preprint. [Version 1] doi: 10.64898/2026.02.10.705189 (PMC12918808; doi:10.64898/2026.02.10.705189)
Supplement: Supplement 1 [file NIHPP2026.02.10.705189v1-supplement-1.pdf]

bioRxiv preprint doi: https://doi.org/10.64898/2026.02.10.705189; this version posted February 11, 2026. The copyright holder for this preprint (which was not certified by peer review) is the author/funder, who has granted bioRxiv a license to display the preprint in perpetuity. It is made available under aCC-BY 4.0 International license.

74. Carmody, S. R., Tran, E. J., Apponi, L. H., Corbett, A. H. & Wente, S. R. The Mitogen-Activated Protein Kinase Slt2 Regulates Nuclear Retention of Non-Heat Shock mRNAs during Heat Shock-Induced Stress. *Mol. Cell. Biol.* **30**, 5168–5179 (2010).

75. Dekker, M., Van Der Giessen, E. & Onck, P. R. Phase separation of intrinsically disordered FG-Nups is driven by highly dynamic FG motifs. *Proc. Natl. Acad. Sci.* **120**, e2221804120 (2023).

76. Patel, S. S., Belmont, B. J., Sante, J. M. & Rexach, M. F. Natively Unfolded Nucleoporins Gate Protein Diffusion across the Nuclear Pore Complex. *Cell* **129**, 83–96 (2007).

77. Krueger, F., James, F., Ewels, P., Afyounian, E. & Schuster-Boeckler, B. FelixKrueger/TrimGalore: v0.6.7 - DOI via Zenodo. Zenodo https://doi.org/10.5281/zenodo.5127899 (2021).

78. Dobin, A. *et al.* STAR: ultrafast universal RNA-seq aligner. *Bioinformatics* **29**, 15–21 (2013).

79. Yu, G., Wang, L.-G., Han, Y. & He, Q.-Y. clusterProfiler: an R package for comparing biological themes among gene clusters. *Omics J. Integr. Biol.* **16**, 284–287 (2012).

80. Fang, F. *et al.* A vector set for systematic metabolic engineering in Saccharomyces cerevisiae. *Yeast Chichester Engl.* **28**, 123–136 (2011).

Supplemental Figures

bioRxiv preprint doi: https://doi.org/10.64898/2026.02.10.705189; this version posted February 11, 2026. The copyright holder for this preprint (which was not certified by peer review) is the author/funder, who has granted bioRxiv a license to display the preprint in perpetuity. It is made available under aCC-BY 4.0 International license.

Figure S1

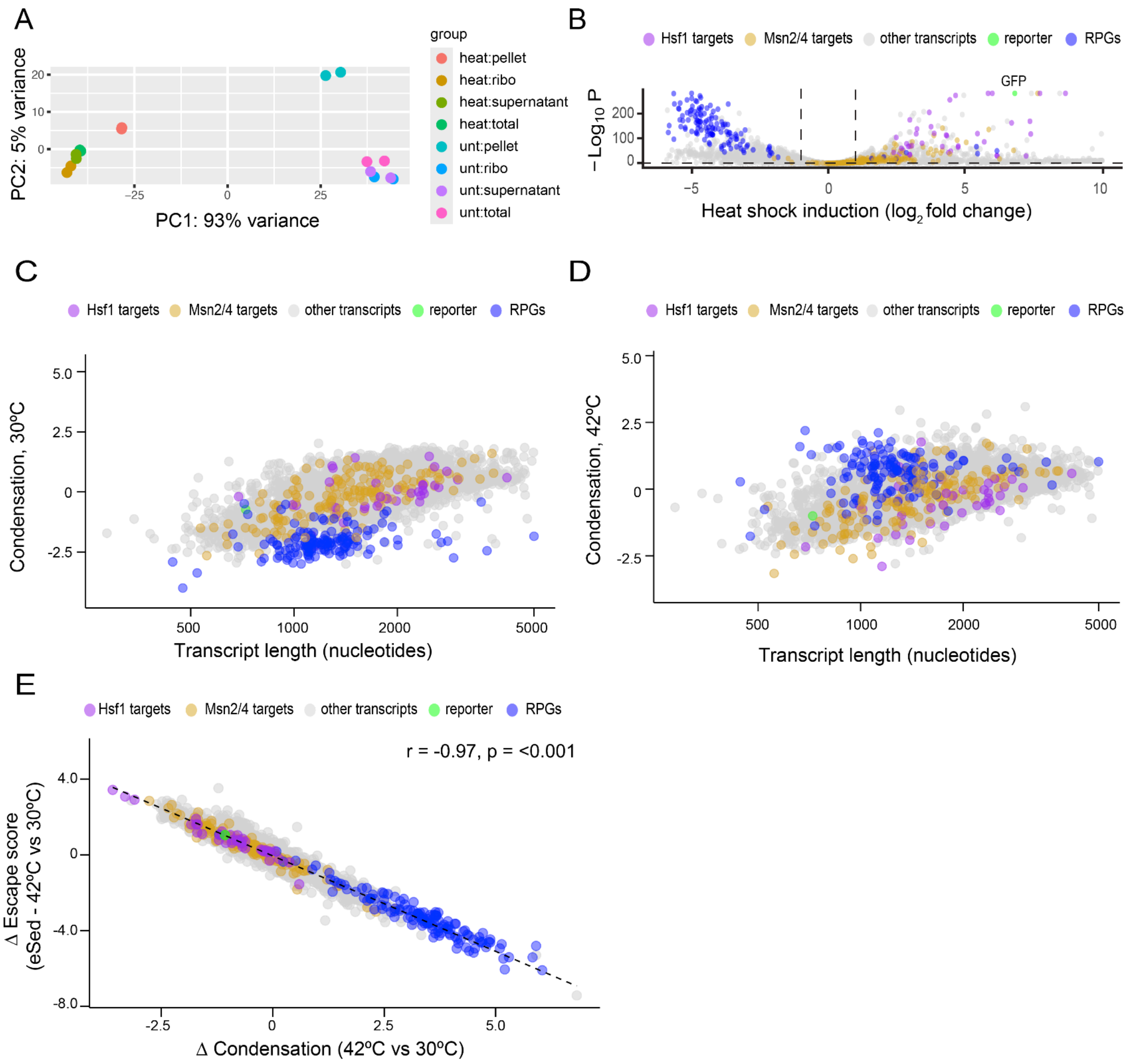

Figure S1. Hsf1 target and RPG transcripts condensation status is independent of length.

A. PCA plot of RNA-Seq libraries by condition (unt = 30℃, heat = 42℃ for 30 minutes) and fraction (pellet = condensate-enriched, ribo = ribosomal, total = unfractionated).

B. Volcano plot of transcriptional changes upon 42℃ heat shock for 30 minutes, showing upregulation of chaperone transcripts (Hsf1 and Msn2/4 targets), and downregulation of ribosomal protein genes (RPG). The Hsf1 synthetic reporter behaves as a canonical Hsf1 target (green).

C. Transcript length versus condensation status at 30℃.

D. Transcript length versus condensation status at 42℃.

bioRxiv preprint doi: https://doi.org/10.64898/2026.02.10.705189; this version posted February 11, 2026. The copyright holder for this preprint (which was not certified by peer review) is the author/funder, who has granted bioRxiv a license to display the preprint in perpetuity. It is made available under aCC-BY 4.0 International license.

E. Scatterplot of length-normalized changes (42℃ versus 30℃) in condensate enrichment with DESeq2 versus proportion in condensate-depleted supernatant fractions with Sed-Seq.

Figure S2

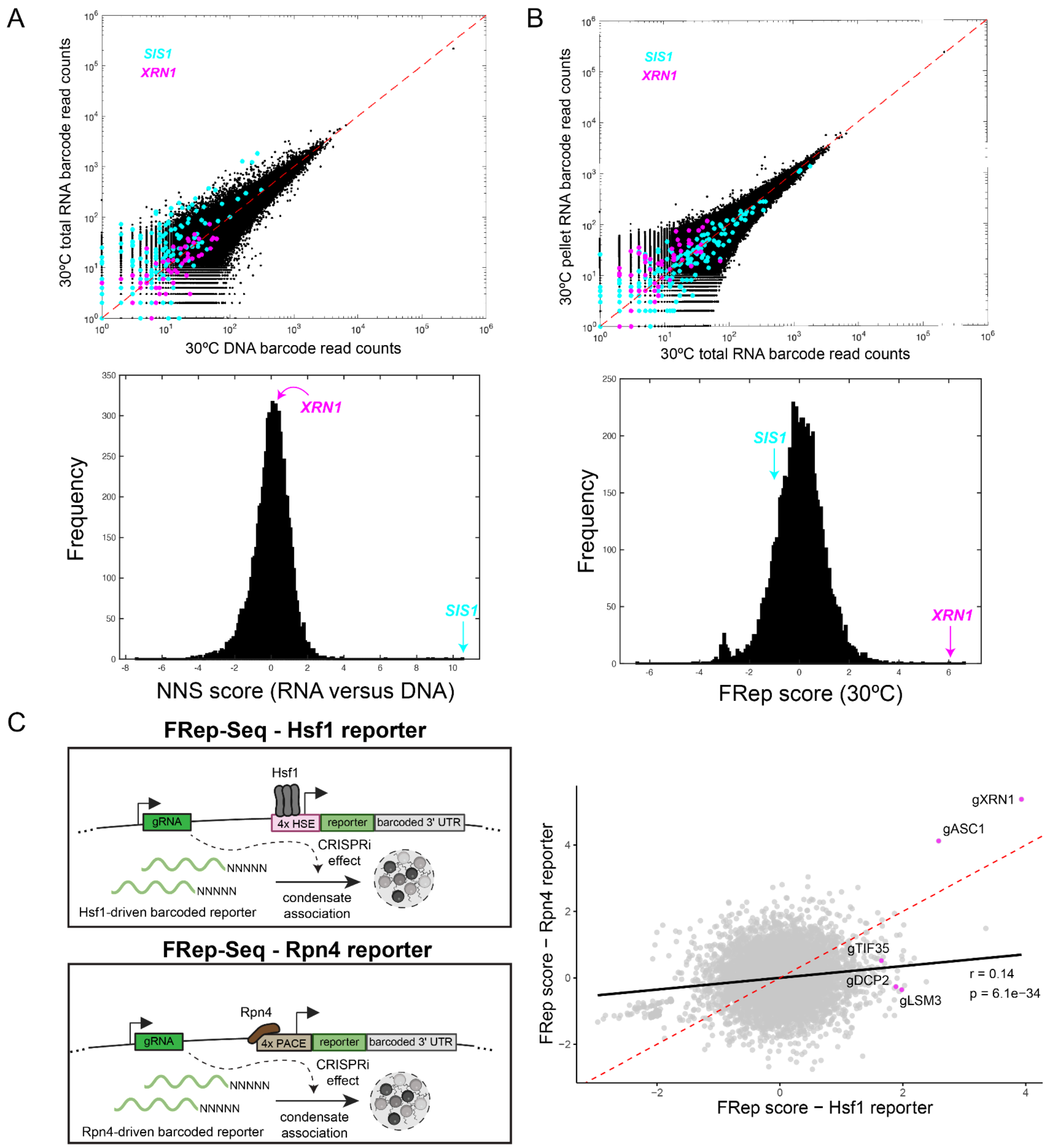

bioRxiv preprint doi: https://doi.org/10.64898/2026.02.10.705189; this version posted February 11, 2026. The copyright holder for this preprint (which was not certified by peer review) is the author/funder, who has granted bioRxiv a license to display the preprint in perpetuity. It is made available under aCC-BY 4.0 International license.

Figure S2. FRep-Seq identifies modulators of condensation despite of transcriptional levels

A. Top panel: scatterplot of unstressed 30℃ plasmid DNA barcode reads versus RNA barcode reads from total unfractionated samples. Every dot represents a unique barcode associated with a given gene. *SIS1* represents the strongest HSR activator identified previously by ReporterSeq[40]. Bottom panel: histogram of calculated NNS scores as a proxy for HSR transcriptional activation.

B. Top panel: scatterplot of unstressed 30℃ RNA barcode reads from total unfractionated versus RNA from condensate-enriched fraction. Bottom panel: histogram of calculated FRep-scores as a proxy for reporter condensation.

C. Left panel: schematics of Hsf1-responsive and Rpn4-responsive reporters and their usage in FRep-Seq. Right panel: scatterplot of FRep-Seq results of reporter condensation for Hsf1-dependent versus Rpn4-dependent reporters at 30℃ steady-state.

bioRxiv preprint doi: https://doi.org/10.64898/2026.02.10.705189; this version posted February 11, 2026. The copyright holder for this preprint (which was not certified by peer review) is the author/funder, who has granted bioRxiv a license to display the preprint in perpetuity. It is made available under aCC-BY 4.0 International license.

Figure S3

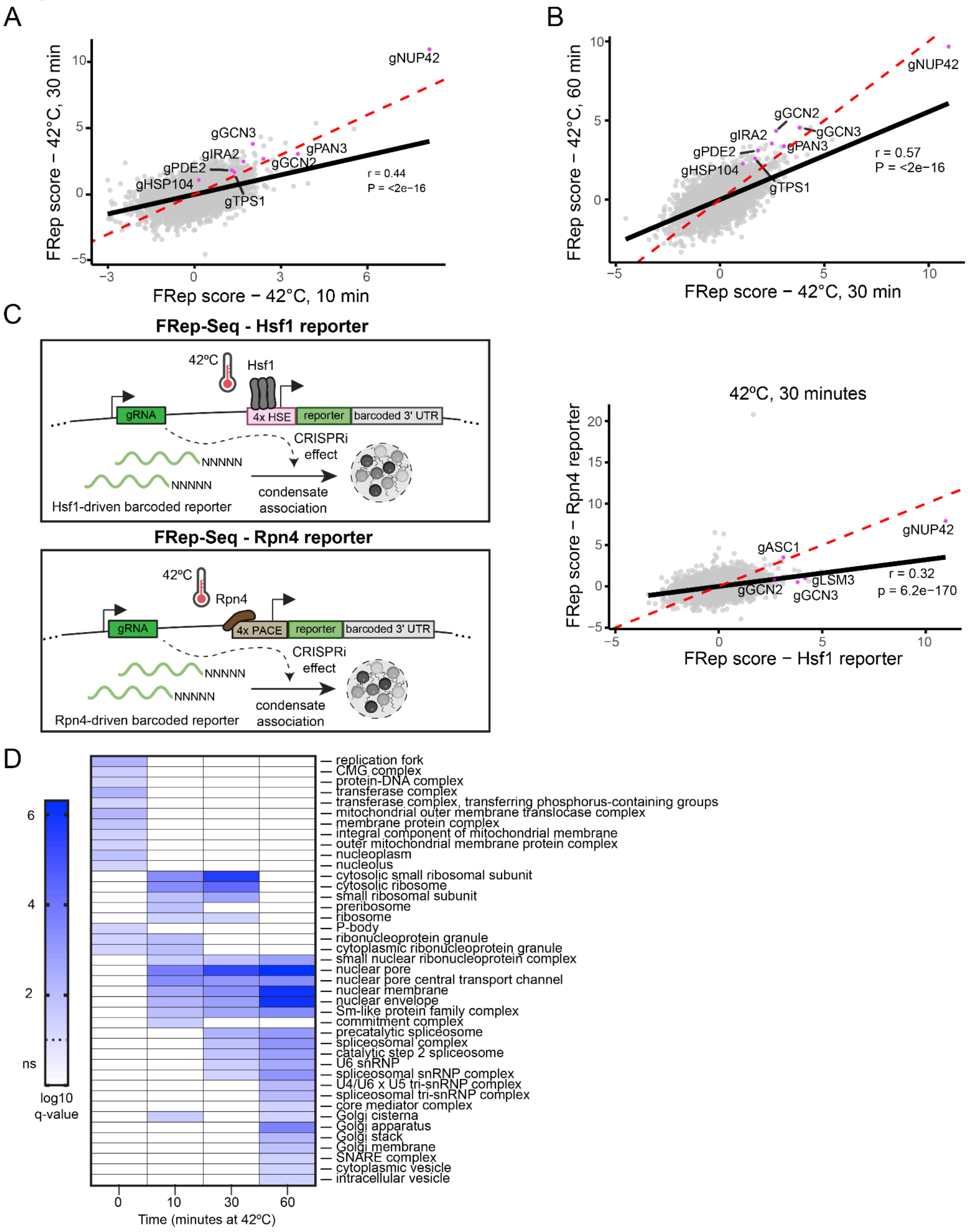

Figure S3. FRep-Seq reveals distinct modulators of Hsf1-reporter escape from condensation at basal and heat shock conditions.

A. Scatterplot of FRep-scores for the Hsf1-responsive reporter at 10 minutes versus 30 minutes of a 42℃ heat shock.

bioRxiv preprint doi: https://doi.org/10.64898/2026.02.10.705189; this version posted February 11, 2026. The copyright holder for this preprint (which was not certified by peer review) is the author/funder, who has granted bioRxiv a license to display the preprint in perpetuity. It is made available under aCC-BY 4.0 International license.

B. Scatterplot of FRep-scores for the Hsf1-responsive reporter at 30 minutes versus 60 minutes of a 42℃ heat shock.

C. Scatterplot of FRep-scores for Hsf1-responsive versus Rpn4-responsive reporter at 42℃, 30 minutes.

D. Gene set enrichment analysis (GSEA) of cellular components enriched among genes whose perturbations increase condensation of the Hsf1 reporter measured by FRep-Seq across all heat shock time points.

bioRxiv preprint doi: https://doi.org/10.64898/2026.02.10.705189; this version posted February 11, 2026. The copyright holder for this preprint (which was not certified by peer review) is the author/funder, who has granted bioRxiv a license to display the preprint in perpetuity. It is made available under aCC-BY 4.0 International license.

Figure S4

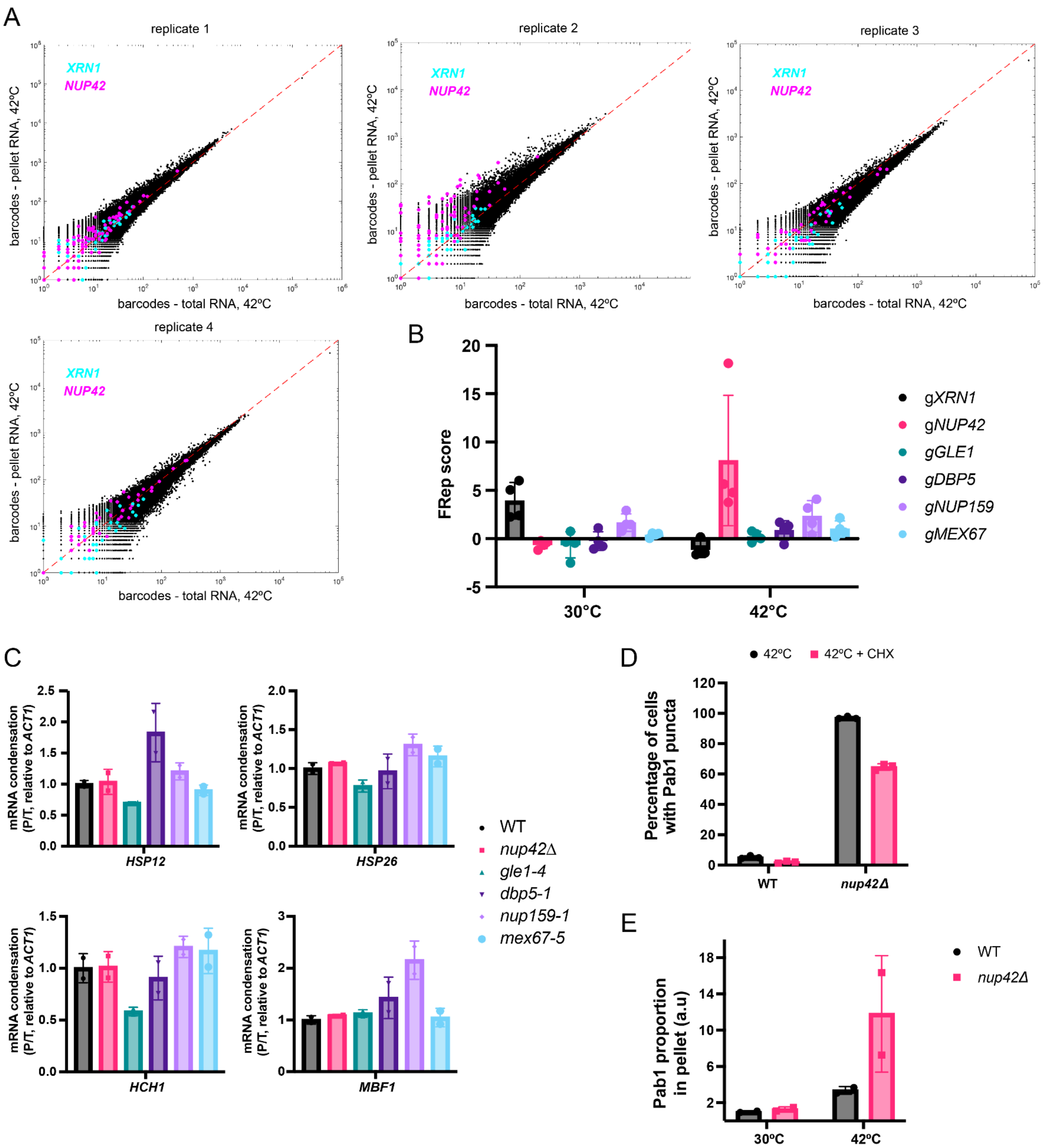

Figure S4. Nup42 does not affect basal condensation.

A. Scatterplots of barcode reads from the total unfractionated sample versus RNA barcode reads condensate-enriched pellet upon 42℃ heat shock for 30 minutes, for all four biological

bioRxiv preprint doi: https://doi.org/10.64898/2026.02.10.705189; this version posted February 11, 2026. The copyright holder for this preprint (which was not certified by peer review) is the author/funder, who has granted bioRxiv a license to display the preprint in perpetuity. It is made available under aCC-BY 4.0 International license.

replicates. Replicates 3 and 4 consist of a different batch of yeast library stocks from another reporter library transformation than replicates 1 and 2.

B. FRep-scores of genes shown in Figure 4B, as well as the strongest hit in the basal unstressed condition, *XRN1*. N = 4 biological replicates.

C. mRNA condensation at 30℃ measured as proportion in pellet relative to total abundance (P/T) for the indicated strains. N = 2 biological replicates.

D. Quantification of fluorescence microscopy images shown in Figure 4G, quantifying percentage of cells with Pab1-mNeonGreen puncta. N = 3 biological replicates, with at least 100 cells quantified per replicate.

E. Quantification of Pab1 proportion in pellet fractions (P/T) from lysates without RNase1 treatment shown in Figure 4H. N = 2 biological replicates.

Figure S5

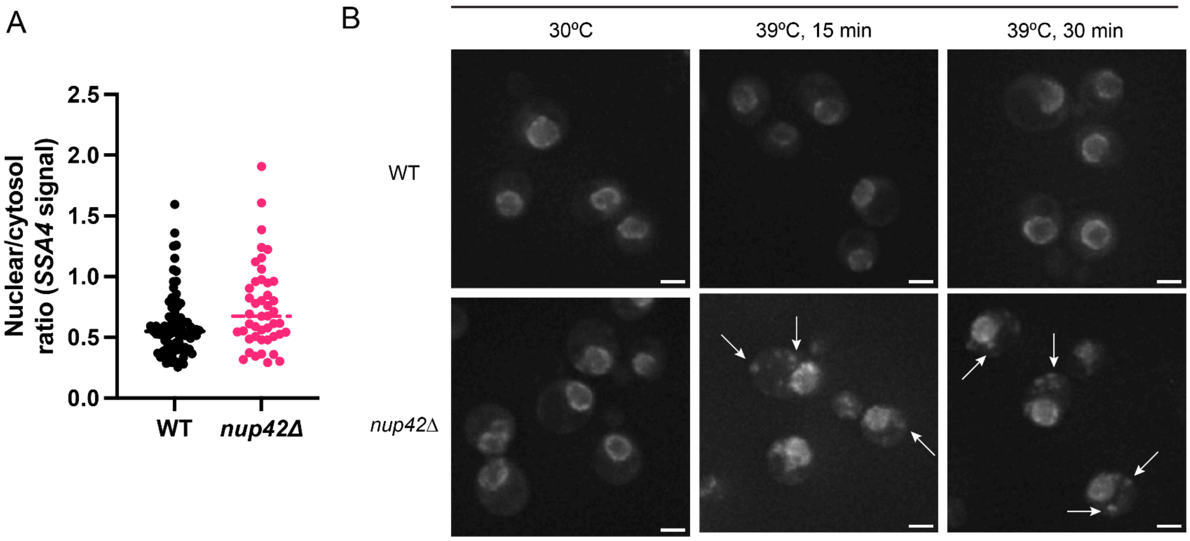

Figure S5. Accumulation of cytosolic Mex67 foci in *nup42Δ* at 39℃.

A. Ratiometric quantification of MCP-NLS-2xGFP in the nucleus relative to cytosol in WT and *nup42Δ* at 42℃, 30 minutes. Quantification of the same images represented in Figure 6B.

B. Representative Z-stack images of WT and *nup42Δ* GFP-Mex67 cells at 30℃ and 39℃ heat shock for 15 and 30 minutes. White arrows indicate cytosolic Mex67 foci. Scale bar = 2 μm.

bioRxiv preprint doi: https://doi.org/10.64898/2026.02.10.705189; this version posted February 11, 2026. The copyright holder for this preprint (which was not certified by peer review) is the author/funder, who has granted bioRxiv a license to display the preprint in perpetuity. It is made available under aCC-BY 4.0 International license.

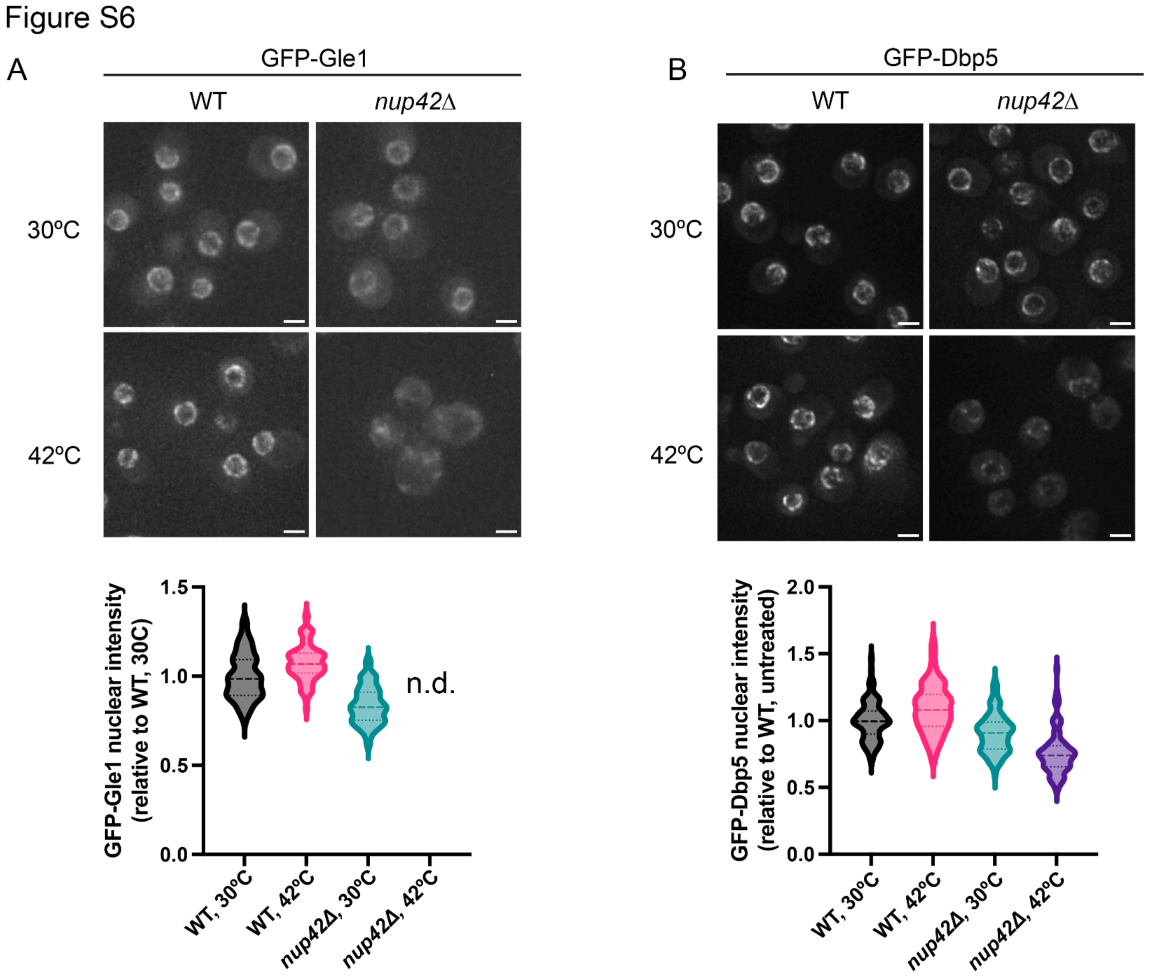

Figure S6. Gle1 and Dbp5 nuclear localization is impaired in the absence of Nup42 during heat shock.

A. Top: representative Z-stack images of WT and *nup42Δ* GFP-Gle1 cells at 30℃ and 42℃, 30 minutes. Scale bar = 2 μm. Bottom: quantification of GFP-Gle1 nuclear intensity of at least 100 individual cells. n.d. = not detected.

B. Top: representative Z-stack images of WT and *nup42Δ* GFP-Dbp5 cells at 30℃ and 42℃, 30 minutes. Scale bar = 2 μm. Bottom: quantification of GFP-Dbp5 nuclear intensity of at least 100 individual cells.

bioRxiv preprint doi: https://doi.org/10.64898/2026.02.10.705189; this version posted February 11, 2026. The copyright holder for this preprint (which was not certified by peer review) is the author/funder, who has granted bioRxiv a license to display the preprint in perpetuity. It is made available under aCC-BY 4.0 International license.

Figure S7

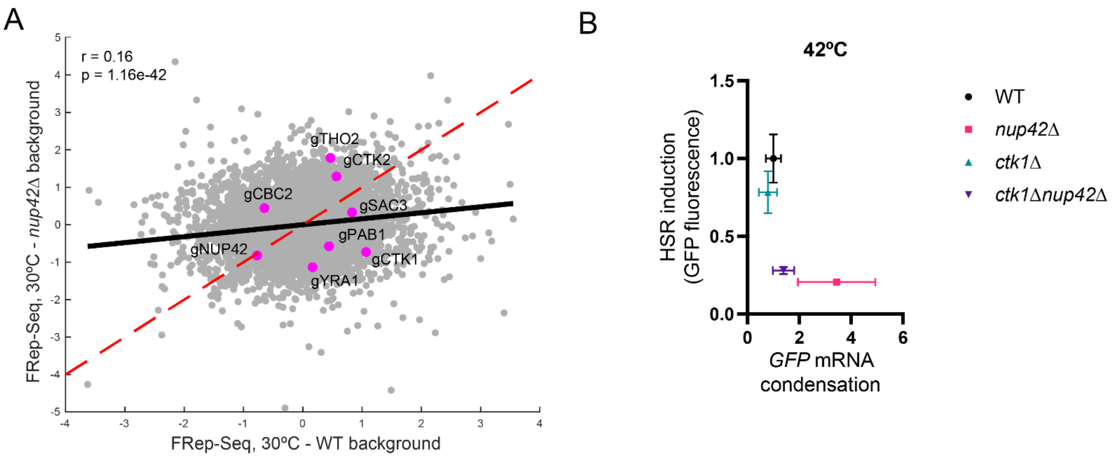

Figure S7. Co-transcriptional mRNP compaction state has minimal effects in mRNP condensation under basal conditions but is a critical determinant in the absence of Nup42 upon heat shock.

A. Scatterplot of FRep-Seq screens conducted in WT versus *nup42Δ* background under basal conditions.

B. Hsf1-responsive reporter transcript condensation versus protein levels upon 42℃ heat shock for 30 minutes, corresponding to data shown in panels 7D and 7E.
